# *Schizophyllum commune* induced genotoxic and cytotoxic effects in *Spodoptera litura*

**DOI:** 10.1038/s41598-018-22919-0

**Published:** 2018-03-16

**Authors:** Mandeep Kaur, Pooja Chadha, Sanehdeep Kaur, Amarjeet Kaur, Rajvir Kaur, Arun Kumar Yadav, Ramandeep Kaur

**Affiliations:** 1 Department of Zoology, Guru Nanak Dev University, Amritsar, India; 2 Departments of Microbiology, Guru Nanak Dev University, Amritsar, India; 3 Department of Biotechnology, Guru Nanak Dev University, Amritsar, India; 4 Department cum National Centre for Human Genome studies and research, Panjab University Chandigarh, Chandigarh, India

## Abstract

In search for ecofriendly alternatives to chemical insecticides the present study was conducted to assess the insecticidal potential of an endophytic fungus *Schizophyllum commune* and its mechanism of toxicity by studying genotoxic and cytotoxic effects as well as repair potential using *Spodoptera litura* (Fabricius) as a model. Different endophytic fungi were isolated and tested for their insecticidal potential against *S. litura*. Among the tested endophytic fungi maximum mortality against *S. litura* was exhibited by *S. commune* isolated from *Aloe vera*. Extended development, reduced adult emergence was observed in larvae fed on diet supplemented with fungal extract. In addition to it the fungus also has propensity to increase oxidative stress which leads to significantly higher DNA damage. The significantly lower frequency of living haemocytes and increased frequency of apoptotic and necrotic cells was also observed in larvae treated with fungal extract. The extent of recovery of damage caused by fungus was found to be very low indicating long term effect of treatment. Phytochemical analysis revealed the presence of various phenolics, terpenoids and protein in fungal extract. Biosafety analysis indicated the non toxic nature of extract. This is the first report showing the insecticidal potential of *S. commune* and the genotoxic and cytotoxic effects associated with it.

## Introduction

Excessive use of insecticides over the years has resulted in the development of insecticide resistant populations of insects. Additionally, these insecticides have many hazardous effects on environment as well as on human health^1^. Thus, there is a need to reduce reliance on chemical insecticides and look for other alternative ecofriendly strategies for the control of insect pests. Biological control of insect pests using microbial insecticides is highly beneficial due to its low cost, specificity and safety to ecosystem^2^. Among the various microorganisms fungal endophytes have been known to impart resistance to insect pests. Fungal endophytes are organisms that grow internally in plant tissues without causing any deleterious effect to host plant^3^. These endophytes produce various bioactive secondary metabolites like alkaloids, flavonoids, phenolic acids and others which have been documented to be responsible for securing their hosts from unfavorable conditions and providing protection from infectious agents^4^. Adverse effects of fungal endophytes on biological parameters of insects have been reported in a number of studies^5,6^. These have been reported to induce mortality, prolong development period, reduce adult emergence as well as cause various morphological deformities in insects^7–11^.

Though a number of studies attributing the effect of fungal bio-pesticides to inhibition of various enzymes and interference with physiological processes are available^12,13^ the effect at cellular and genetic level has not been explored. The level of DNA damage after treatment with fungal metabolites would be related to the ability of a pest to survive and reproduce. Impaired activity of antioxidant defense and DNA repair contribute to the DNA damage by free radicals. Previously the effect of radiations on the genetic material for the purpose of pest management has been evaluated on *Plodia interpunctella* (Hubner)^14^, *Curculio sikkimensis* (Heller)^15^, *Plutella xylostella* (Linnaeus)^16^, *Liriomyza trifolii* (Burgess)^17^, and *Spodoptera litura* (Fabricius)^18^ but no studies are available which show the impact of microbial bio-pesticides up to the DNA level. In the present study genotoxicity of fungal metabolites was assessed by using comet assay. Among the various techniques of genotoxicity testing, the use of comet assay or Single cell gel electrophoresis (SCGE) assay is considered as a simple, quick and inexpensive method. It allows the detection of single and double strand breaks, oxidative base damage and DNA cross linking^19^ as well as assesses the repair potential. So, in the present study attempts have been made to analyze the oxidative stress, cytotoxicity and genotoxicity as well as repair of damage by fungal extract using *S. litura* as a model.

*S. litura* is one of the economically important pest with wide host range of more than 180 plant species^20^. It has been reported to cause huge crop losses to a number of agriculturally important crops like cabbage, soybeans, cotton, and many other vegetables^21^. Chemical control is the main strategy being adopted by the farmers which has resulted in development of resistant populations of insects to a large number of insecticides^22^.

Keeping this in view the present investigation has been undertaken to isolate and screen endophytic fungi for their insecticidal potential against *S. litura*. Oxidative stress, cytotoxicity and genotoxicity induced by ethyl acetate fungal extract were also assessed. Further recovery of all the parameters was ascertained. Biosafety analysis of the selected isolate was also determined.

## Results

### Isolation and Screening results

Fifteen different fungi were isolated from different plants and screened for their insecticidal activity. Maximum mortality exhibiting A4 culture (Table 1) was selected for further studies.

**Table 1:** Screening of fungi isolated from different plants for insecticidal potential against *S. litura*.

Cultures	Larval mortality (%) (Mean ± S.E)
Control	13.33 ± 4.22^a^
‘T1(Tulsi leaf)	23.33 ± 6.15^ab^
T2(Tulsi leaf)	46.66 ± 6.66^bc^
T3(Tulsi leaf)	30.00 ± 4.47^ab^
T4(Tulsi leaf)	16.66 ± 6.15^ab^
A1(Aloevera leaf)	20.00 ± 7.30^ab^
A4(Alovera leaf)	63.33 ± 9.54^c^
AL(Amaltas leaf)	30.00 ± 6.83^ab^
AS(Amaltas stem)	26.67 ± 8.43^ab^
F value	5.357**

Values are Mean ± S.E. Significance ascribed as *p < 0.05 and **p < 0.01 (One way ANOVA). The values followed by different letters a, b, c within the column are significantly different (Tukey’s test, p ≤ 0.05).

### Identification of fungus

**Figure 1:**
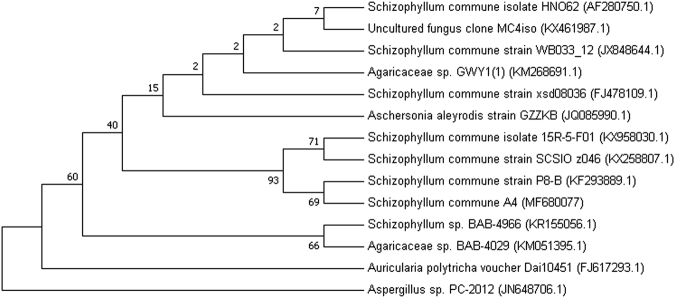
Phylogenetic tree showing the position of A4 on the basis of ITS1-5.8 rDNA-ITS2 gene sequence analysis. The evolutionary history was inferred using the Neighbor-Joining method. The bootstrap consensus tree inferred from 1000 replicates is taken to represent the evolutionary history of the taxa analyzed. The percentage of replicate trees in which the associated taxa clustered together in the bootstrap test (1000 replicates) are shown next to the branches. The evolutionary distances were computed using the Kimura 2-parameter method and are in the units of the number of base substitutions per site. The analysis involved 14 nucleotide sequences. Codon positions included were 1st + 2nd + 3rd + Noncoding. All positions containing gaps and missing data were eliminated. There were a total of 521 positions in the final dataset. Evolutionary analyses were conducted in MEGA7.

Fungus was identified on morphological and molecular basis. The fungus was rapid growing and produced white cottony colonies on Potato Dextrose Agar (PDA). Microscopic observations revealed branched and septate hyphae. For molecular identification the DNA of the selected fungus was isolated and amplified using ITS1-5.8S-ITS2 region. The length of the amplified sequence was found to be 616 bp.The sequence was deposited into GeneBank under accession number: MF680077. The sequence similarity of obtained sequence was matched with other available databases from NCBI database using BLAST and a phylogenetic tree was constructed using MEGA7 program (Fig. 1). The strain was found to show 100% similarity with *Schizophyllum commune* and phylogeny tree showed that the A4 fungus belong to genera *Schizophyllum* and species of *S. commune*. On the basis of molecular and morphological characteristics the culture was identified as *S. commune*.

### Bioassay studies

Bioassay studies have confirmed the detrimental effect of fungus A4 (*S. commune*) on *S. litura*. Artificial diet supplemented with ethyl acetate extract of fungus *S. commune* induced 40.00–73.33% mortality in larvae of *S. litura*. With increase in concentration of the extract mortality rate has increased significantly as compared to control. The highest concentration resulted in 7.333 times higher mortality than control (F = 13.533, p < 0.01) as shown in Table 2. The LC50 value was found to be 276.542 µg/ml.

**Table 2:** Mortality of *S. litura* larvae fed on diet supplemented with different concentrations of ethyl acetate extract of *S. commune*.

Concentrations (µg/ml)	Larval Mortality (%) (Mean ± S.E)
Control	10.00 ± 4.47^a^
125	40.00 ± 7.30^b^
250	46.66 ± 8.43^bc^
500	60.00 ± 5.16^bc^
1000	66.66 ± 4.21^bc^
2000	73.33 ± 6.66^c^
F value	13.533**

The values are mean ± standard error. Significance ascribed as *p < 0.05 and **p < 0.01 (One way ANOVA). Different letters a, b, c within the column are significantly different (Tukey’s test, p ≤ 0.05) and signify the effect of concentration.

As evident from the Table 3 addition of higher concentration i.e 1000 and 2000 µg/ml significantly extended the larval period with respect to control (F = 5.620, p < 0.01), however no significant effect was detected on pupal period except for highest concentration (F = 3.249, p < 0.05). The overall development period seems to be increased but the significant increase was recorded at 2000 µg/ml (F = 7.128, p < 0.01).

**Table 3:** Influence of ethyl acetate extract of *S. commune* on development of *S. litura*.

Concentration (µg/ml)	Larval period(days) (Mean ± S.E)	Pupal period(days) (Mean ± S.E)	Total development period(days) (Mean ± S.E)
Control	22.59 ± 1.52^a^	10.58 ± 0.43^ab^	33.17 ± 1.49^a^
125	23.46 ± 1.72^a^	9.87 ± 1.08^ab^	33.34 ± 2.01^a^
250	23.77 ± 1.30^ab^	10.45 ± 0.61^ab^	34.22 ± 1.35^a^
500	26.17 ± 0.98^abc^	9.83 ± 0.87^ab^	36.00 ± 0.63^a^
1000	30.08 ± 2.06^bc^	8.66 ± 0.56^a^	38.75 ± 2.03^ab^
2000	30.90 ± 1.22^c^	12.66 ± 0.65^b^	43.56 ± 1.04^b^
F value	5.620**	3.249*	7.128**

The values are mean ± standard error. Significance ascribed as *p < 0.05 and **p < 0.01 (One way ANOVA). Different letters a, b, c within the columns are significantly different (Tukey’s test, p ≤ 0.05) and signify the effect of concentration.

Furthermore, the adverse effects of fungal extract have also been observed on adult emergence. The percentage of adult emergence was found to be decreased from 90.00 to 33.33% due to ingestion of ethyl acetate extract of *S. commune* by larvae (F = 2.870, p < 0.05) as shown in Table 4.

**Table 4:** Influence of ethyl acetate extract of *S. commune* on percentage of adult emergence of *S. litura*.

Concentration (µg/ml)	Percentage of Adult emergence (%) (Mean ± S.E)
Control	90.00 ± 6.83^ab^
125	72.21 ± 10.90^ab^
250	44.40 ± 14.04^ab^
500	66.66 ± 10.54^ab^
1000	66.66 ± 10.54^ab^
2000	33.33 ± 16.66^b^
F value	2.870*

The values are mean ± standard error. Significance ascribed as *p < 0.05 and **p < 0.01 (One way ANOVA). Different letters a, b within the column are significantly different (Tukey’s test, p ≤ 0.05) and signify the effect of concentration.

### Effect of ethyl acetate extract of *S. commune* on lipid peroxidation

Table 5 revealed the effect of treatment with ethyl acetate extract of *S. commune* on lipid peroxidation as indicated by malondialdehyde (MDA) content in haemolymph of *S. litura* at different hours of exposure. A non-significant (p > 0.05) change in MDA content at 24 hr exposure group was observed while a significant (p < 0.05) increase in MDA content was observed in 48 hr,72 hr and 96 hr exposure groups with respect to control. The effect of duration of exposure was also found to be significant (p < 0.05).

**Table 5:** MDA content in haemolymph of *S. litura* after treatment with ethyl acetate extract of *S. commune*.

Parameter	Group	Exposure Time (hrs)			
		24	48	72	96
MDA Content	Control	5.65 ± 0.06^a^	5.83 ± 0.11^a^	5.79 ± 0.07^a^	6.04 ± 0.09^a^
	EG(LC50)	6.29 ± 0.22^a^	7.40 ± 0.19*^b^	8.07 ± 0.08*^b^	9.21 ± 0.09*^c^

EG = Exposed group. The values represented as mean ± standard error. *Ascribes the significant difference between exposed group and control group (t-test, p ≤ 0.05). Different letters a, b, c between the columns are significantly different (Tukey’s test, p ≤ 0.05) and signify the effect of duration.

### Effect of ethyl acetate extract of *S. commune* on cell viability

**Figure 2:**
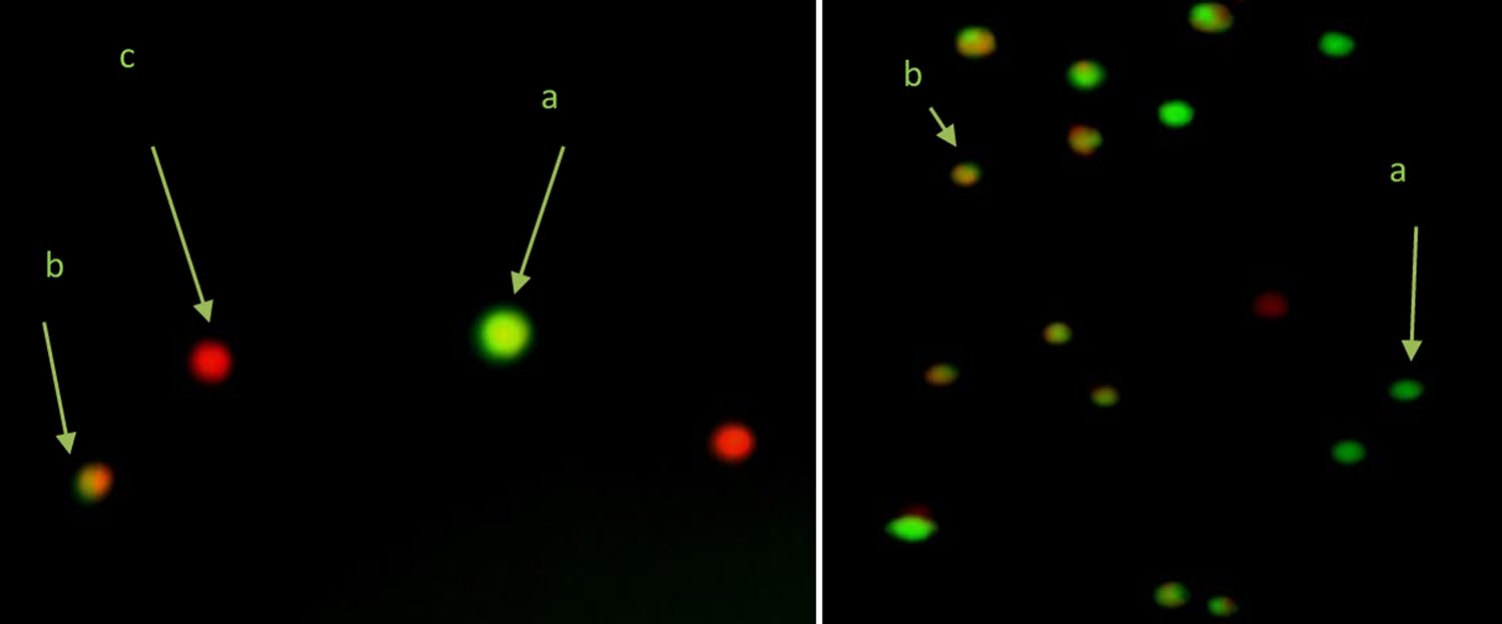
Typical morphological changes of haemocytes induced by ethyl acetate extract of *S. commune*, stained with AO/EB. The images were taken using fluorescence microscopy at 400 ×. (**a**) Living cells (nuclei staining showing green chromatin with organized structures); (**b**) apoptotic cells (condensed or fragmented chromatin showing green and red color); (**c**) necrotic cells (nuclei staining similar as live cells except the chromatin is red in color instead of green).

The data summarized in Table 6 showed living cells, apoptotic cells and necrotic cells percentage obtained with Acridine Orange/Ethidium Bromide (AO/EB) double staining of haemocytes of *S. litura* exposed to fungal extract amended diet at different time intervals. A significant (p < 0.05) decrease in living cells percentage while a significant(p < 0.05) increase in apoptotic cells and necrotic cells percentage was observed in larvae exposed to fungal extract as compared to their respective controls (t-test) at each time interval. Further to determine the effect of duration of exposure, ANOVA followed by post hoc Tukey’s test was carried out. It was found that the values of living cells percentage for all exposed groups increased significantly (p < 0.05) up to 72 hours and then decreased. Although, the overall effect of time duration was found to be significant (p < 0.05) in case of apoptotic cells percentage, however, a corresponding increase in percentage of apoptotic cells was not found with increase in time duration. Highest apoptotic cells percentage was observed after a 24 hr of exposure. In case of necrotic cells percentage, a time dependent significant (p < 0.05) increase in induction of necrosis was observed. The images of living cells, apoptotic cells and necrotic cells obtained after AO/EB double staining of haemocytes of *S. litura* are shown in Fig. 2.

**Table 6:** Living cells, Apoptotic cells and Necrotic cells percentage in haemolymph of *S. litura* after treatment with ethyl acetate extract of *S. commune*.

Parameters	Groups	Exposure Time (hrs)			
		24	48	72	96
Living Cells Percentage	Control	85.25 ± 1.88^a^	83.50 ± 0.87^a^	84.75 ± 1.29^a^	84.50 ± 0.86^a^
	EG(LC50)	46.50 ± 1.15*^a^	53.00 ± 1.73*^ab^	56.50 ± 2.59*^b^	49.75 ± 0.72*^ab^
Apoptotic Cells Percentage	Control	10.00 ± 1.15^a^	12.75 ± 0.14^a^	10.25 ± 0.43^a^	10.75 ± 1.01^a^
	EG(LC50)	47.50 ± 1.44*^c^	37.00 ± 1.15*^b^	30.00 ± 0.29*^a^	32.25 ± 1.88*^ab^
Necrotic Cells Percentage	Control	4.75 ± 0.72^a^	3.75 ± 0.72^a^	5.00 ± 0.87^a^	4.75 ± 0.14^a^
	EG(LC50)	6.00 ± 0.28^a^	10.00 ± 0.57*^ab^	13.50 ± 2.31*^bc^	18.00 ± 1.15*^c^

EG = Exposed group. The values represented as mean ± standard error. *Ascribes the significant difference between exposed group and control group (t-test, p ≤ 0.05). Different letters a, b, c between the columns are significantly different (Tukey’s test, p ≤ 0.05) and signify the effect of duration.

### Effect of ethyl acetate extract of *S. commune* on DNA damage

Table 7 revealed the genotoxic effect of fungal extract following treatment with ethyl acetate extract of *S. commune* at different hours of exposure using comet assay. The parameters used to assess genotoxicity were Percent Tail DNA and Olive Tail Moment (OTM).The nuclei with a clear tail like extension were observed after treatment indicating that the cells of insect were damaged and DNA strands were also broken. A typical rounded nuclei was observed in control while maximum length of tail was formed after treatment at 96 hrs of exposure. A significant (p < 0.05) increase in Percent Tail DNA and OTM values was observed in exposed groups as compared to their respective controls at all time intervals. The effect of duration of exposure was also found to be significant (p < 0.05). At 72 hour of exposure the values decreased slightly but again at 96 hour of exposure the values of Percent Tail DNA and OTM were found to be increased.

**Table 7:** Percent Tail DNA and OTM in haemocytes of *S. litura* after treatment with ethyl acetate extract of *S. commune*.

Parameters	Groups	Exposure Time (hrs)			
		24	48	72	96
Percent Tail DNA	Control	5.54 ± 0.38^a^	5.49 ± 0.35^a^	5.09 ± 0.41^a^	5.39 ± 0.28^a^
	EG(LC50)	10.02 ± 0.51*^a^	21.60 ± 1.77*^b^	20.35 ± 1.91*^b^	29.53 ± 1.88*^c^
OTM	Control	4.01 ± 0.46^a^	4.58 ± 0.88^a^	3.13 ± 0.05^a^	4.36 ± 0.25^a^
	EG(LC50)	6.55 ± 0.58*^a^	17.63 ± 0.46*^b^	15.28 ± 1.18*^b^	17.77 ± 3.18*^b^

EG = Exposed group. The values represented as mean ± standard error. *Ascribes the significant difference between exposed group and control group (t-test, p ≤ 0.05). Different letters a, b, c between the columns are significantly different (Tukey’s test, p ≤ 0.05) and signify the effect of duration.

### Recovery of DNA damage

**Figure 3:**
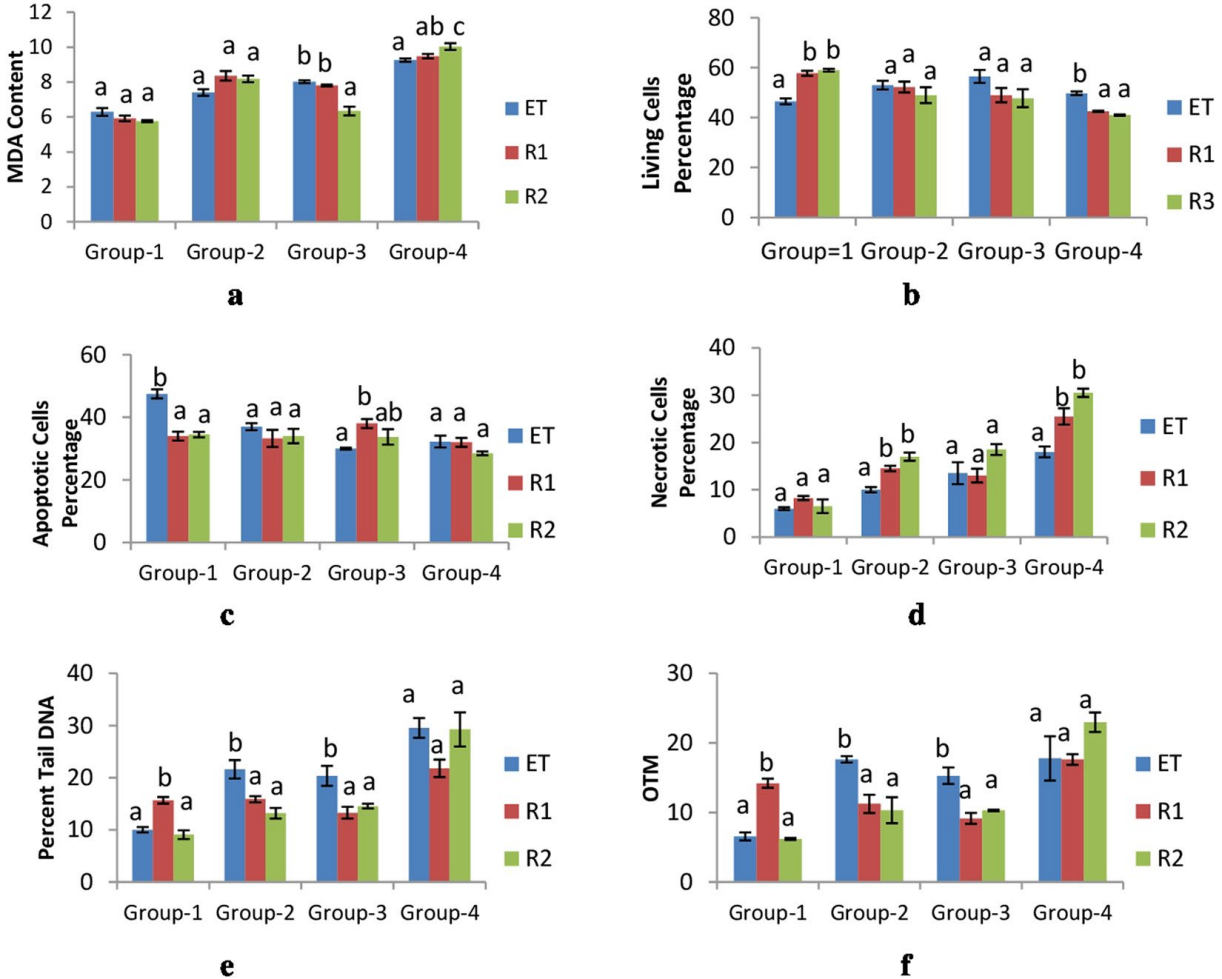
(**a**–**f**) Represent the MDA content, Living Cells, Apoptotic Cells and Necrotic Cells Percentage, Percent Tail DNA and OTM in different exposure time and recovery groups.Group-1 = Exposure Time 24 hr, Group-2 = Exposure Time 48 hr, Group-3 = Exposure Time 72hr, Group-4 = Exposure Time 96 hr, ET = Exposure time, R1 = Recovery after 1 day, R2 = Recovery after 2 days. Different letters a, b, c, signify the difference between exposure time group and recovery groups. Error bars represent the standard error. The same letters indicate homogenous groups.

Recovery of DNA damage, MDA content and cell viability was assessed in both recovery groups (R1 and R2) and compared with their respective exposure time groups. The results have been summarized in Fig. 3(a–f).

*S. litura* larvae exposed to fungal extract for 24 hr and 48 hr did not show any significant (p > 0.05) change in MDA content in recovery groups as compared to their respective exposure time group. However 72 hr exposure time group showed a significant (p < 0.05) decrease in MDA content in recovery groups as compared to its exposure time group. 96 hr exposure time group showed a significant (p < 0.05) increase in MDA content in both recovery groups with respect to its exposure time group. So it is clearly indicated that the level of lipid peroxidation as indicated by value of MDA content did not get lowered after feeding larvae to control diet (Fig. 3a).

Figure 3(b–d) summarize the results of Living Cells Percentage, Apoptotic Cells Percentage and Necrotic Cells percentage in recovery groups of each time exposure as compared to their respective exposure time group. For 24 hr exposure time group the value of living cells percentage was found to be increase significantly (p < 0.05) and the value of apoptotic cells percentage was decreased significantly (p < 0.05) and the value of necrotic cells increased but non-significantly (p > 0.05). Perusal of the Fig. 3(b–d) clearly indicated that for the three parameters of cell viability testing, no recovery was observed. Living cell percentage was found to be low in recovery groups while the necrotic cells percentage was found to be significantly (p < 0.05) high in recovery groups. So in overall apoptosis and necrosis was not getting lowered after giving control diet to larvae which were fed with fungal extract amended diet.

Figure 3(e–f) reveal the results of recovery of DNA damage. The values showed variable trend in the recovery groups for both the parameters. The values of %Tail DNA and OTM increased significantly (p < 0.05) after 1 day of recovery and then decreased after 2days of recovery for 24 hr exposure time group. however for 48 hr and 72 hr exposure time groups the values of %Tail DNA and OTM significantly (p < 0.05) decreased in both recovery groups as compared to their respective exposure time groups. For 96 hr exposure time group the values of both parameters did not found to be differ significantly (p > 0.05) in recovery groups as compared to exposure time group.

### Phytochemical detection results

**Figure 4:**
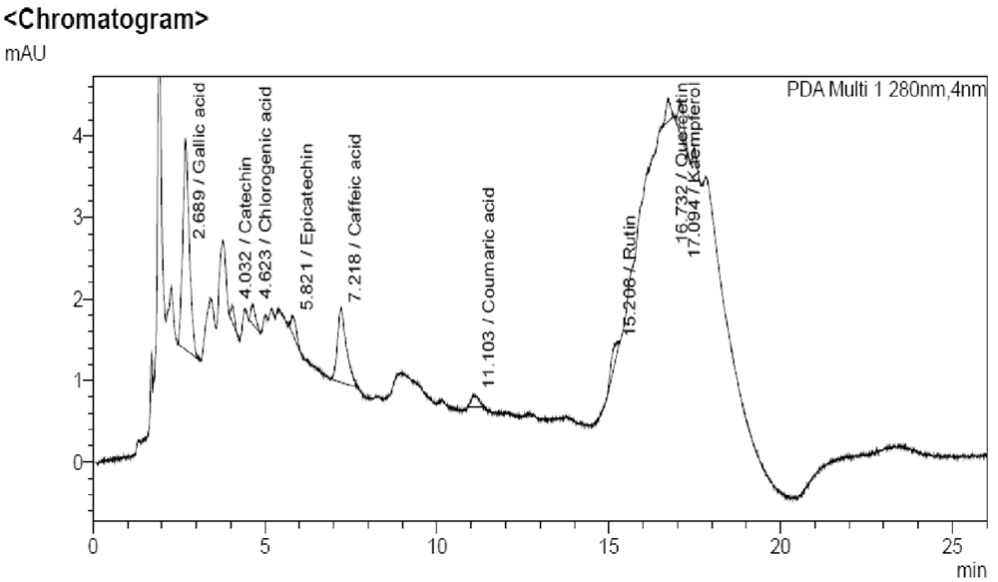
UHPLC chromatogram of the ethyl acetate extract of *S. commune* detected at 280 nm.

Phytochemicals tests revealed the presence of gylcosides, proteins, flavanoids, triterpenoids and phenolics in the ethyl acetate extract of *S. commune*. Folin-Ciocalteu assay determined the Total Phenolic Content (TPC) which was equivalent to 870 µg/ml of gallic acid. Ultra-high performance liquid chromatography (UHPLC) analysis confirmed the presence of gallic acid, catechin, chlorogenic acid, epicatechin, caffeic acid, coumaric acid, rutin, quercetin and kaempferol phenolic compounds in ethyl acetate extract of fungus *S. commune* (Fig. 4).

### Effect of ethyl acetate extract of *S. commune* on Chinese Hamster Ovary (CHO) cell lines

**Figure 5:**
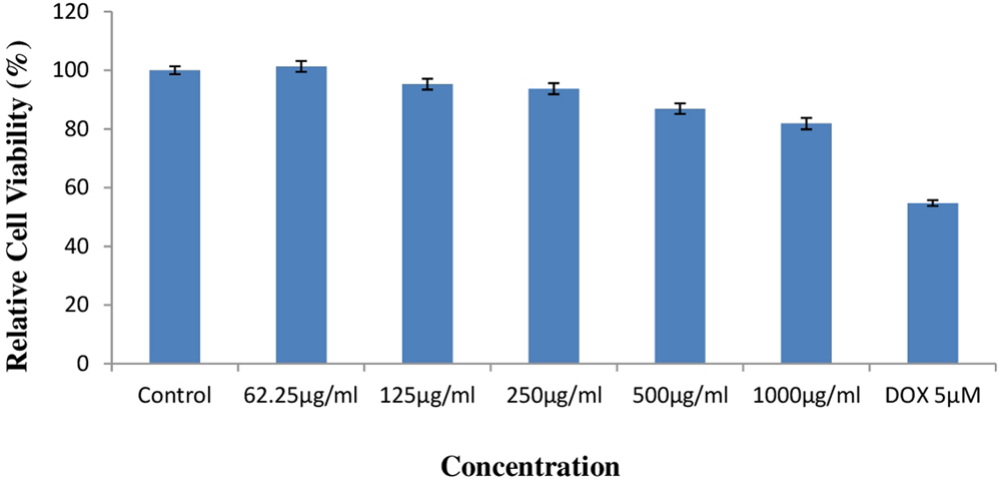
Effect of ethyl acetate extract of *S. commune* on Relative cell viability of CHO cell lines after treatment with different concentrations of ethyl acetate extract of fungus.

To find out the effect of fungal extract on mammals MTT assay on CHO cell lines was carried out. The data is summarized in Fig. 5 which revealed the effect of treatment with different concentrations of *S. commune* fungal extract on Relative cell viability. Relative cell viability of control was found to be 100% and after treatment with highest concentration of fungal extract it was found to be 81.82% which is very much higher as compared to doxorubicin (DOX) a cytotoxic drug treated cells. So *S. commune* fungal extract is proved to be safe for mammalian cell lines showing negligible effect on CHO cell lines.

## Discussion

Within the last two decades endophytes have attracted greater attention due to their capabilities to protect their host from pest and pathogens. Studies have concluded that plants inoculated with endophytic fungi showed resistance against insect pests as compared to non inoculated plants. Prior inoculation of tomato plants with an endophytic fungi *Acremonium strictum* was found to decrease the performance of the pest cotton bollworm (*Helicoverpa armigera*) (Hubner)^23^. Similarly inoculation of squash plants with an endophytic strain of *Fusarium oxysporum* is reported to provide protection from *Aphis gossypii* (Glover)^24^. Retarded development was also observed in insects fed on endophytes inoculated plants^25^. Endophytic fungi *Paecilomyces* sp. and *Cladosporium* sp. isolated from leaves of *Nerium oleander* were evaluated as biocontrol agents against bean weevil *Acanthoscelides obtectus* (Say)^26^. On a wide variety of crops, fungal endophytes have been reported to deter feeding, oviposition and performance of stem boring, sap sucking, chewing and leaf mining insects^27^. On the other hand endophytic fungi are also used as a potential candidate for systemic delivery of biopesticides to the host plant without direct manipulation of plant genome and can improve anti-insect activity of the plant. Zhao *et al*.^28^ used recombinant fungus to control sap sucking pest. Qi *et al*.^29^ developed a pest management strategy to control sap sucking insect using recombinant endophytic fungi *Chetomium globosum* expressing *Pinellia ternate* agglutinin.

In the present study endophytic fungi were isolated from various plants and their insecticidal activity was evaluated against *S. litura*. Maximum mortality was evinced by culture A4 identified as *Schizophyllum commune*. *S. commune* is basidiomycetes belonging to the schizophyllaceae family of order agaricales. It is a cosmopolitan species and wood rot basidiomycetes^30^. It is also popular as an edible mushroom in some regions eg. Peninsular Malaysia^31^ and Mexico^32^. It has been found to be endophyte of *Musa* spp., *Tectona grandis* and *Panax ginseng* etc.^33–35^.

Various bioactivities like antibacterial^36^ and antifungal^37,38^ have been reported from of *S. commune*. It has been reported to produce some volatile organic compounds (VOCs) having nematicidal and nematostatic effects^39^.Though various activities shown by *S. commune* were reported earlier but insecticidal properties of this fungus have not been described yet. So to the best of our knowledge this is the first report indicating its insecticidal potential.

It was observed that the artificial diet supplemented with different concentrations of ethyl acetate extract of *S. commune* exerted negative effect on larval survival. All concentrations showed significantly higher mortality than control. The fungal extract was found to extend the development period and reduce the emergence of *S. litura* adults. Recently few studies have documented the insecticidal activity of endophytic fungi viz *Alternaria* sp., *Cladosporium* sp. and *Nigospora* sp. on *S. litura*^11,40–42^. Some endophytic fungi *Aspergillus flavus, Nigrospora* sp*. and Comothyrium* sp. showed adverse effects on life cycle of *Aphis gossypii* (Glover)^43^. *S. commune* has been reported to produce secondary metabolites like pthalic acid and triterpenoid saponin^3,36^. There are some reports which showed insecticidal activity of pthalic acid derivatives. Phthalic acid diamides derivatives have been reported to show insecticidal activities against *Pseudaletia separate* (Walker), *Plutella xylostella* (Linnaeus.), *Mythimna separate* (Walker)*, Spodoptera exigua* (Hubner)^44,45^. Triterpene saponin was also been found to exert negative impact on *Acyrthosiphon pisum* (Harris) and on some other insects^46,47^. In the present study phytochemical screening of extract showed the presence of phenolics. Previous literature has evinced the insecticidal activity of few phenolic compounds like gallic acid, rutin, quercetin, chlorogenic acid etc^48,49^. So the high amount of phenolics present in the extract could also be responsible for insecticidal activity.

To ascertain the cause of high mortality and abnormal development of *S. litura* after treatment with fungal extract further observations at cellular and genetic levels were made. The DNA damage and repair as well as cell viability and induced oxidative stress in the exposed group were studied. MDA content in fungal extract treated larvae was found to be significantly higher than control and also increase was time dependent. Similarly increased oxidative stress was observed in fat body and midgut of *Galleria mellonella* (Linnaeus) when fed with diet supplemented with boric acid^50^. Earlier comparison of MDA contents in larvae and pupae stage of *S. litura* after treatment with xenobiotic compounds were done by Huang *et al*.^51^ and the MDA content in larvae stage was found to be four times higher than non feeding pupal stage.

The consumption of fungal extract by larvae also significantly reduced the percentage of living cells and increased the percentage of apoptotic and necrotic cells showing the cytotoxic effect of fungal extract. Different researchers have evaluated the cytotoxic and apoptotic effects of different plants as well as fungal extracts on various cell lines^52–55^. Fernando *et al*.^56^ evaluated the antioxidant potential, invitro cytotoxicity and apoptotic effect induced by crude extract of terrestrial macrofungus *Anthracophyllum lateritium* against RD sarcoma cell line. *In vivo* cytotoxic effects of venom from endoparasitoid *Pimpla turionellae* on haemocytes of host *G.mellonella* (Linnaeus) was studied by Er *et al*.^57^.

Further the comet assay studies showed significantly increased DNA damage in fungal extract treated groups as compared to control. Results demonstrated that with increase in time duration DNA damage increased in exposed groups. Very few studies have been reported on induction of DNA damage in insects^58–60^. DNA damage in hemocytes of insect was assessed after exposure to food contaminated with cadmium and lead by Yousef *et al*.^19^ and it was found that cadmium and lead caused large scale DNA damage in insect. Very few studies examined DNA damage in context of biocontrol^18,61^. Shetty *et al*.^62^ evaluated the gamma radiation induced DNA damage in *Aedes aegypti* using comet assay. All these studies used insect as a model to check toxicity of pollutants and other toxic chemicals.

In addition to DNA damage DNA repair was also studied in the exposure groups after feeding the control diet for 1 day and 2days. Other parameters viz oxidative stress and cytotoxicity was also measured in repair groups and compared with their respective exposure time groups. It is clear from the results that the lipid peroxidation profile and cytotoxicity was also not decline in recovery groups as well as the DNA damage have not showed any repair in the all recovery groups. Low level of DNA damage was partially repaired but high DNA damage was not repaired. DNA damage and repair studies are more prominent in vertebrates; invertebrates are less explored in context of this. Recently one study examined the susceptibility to oxidative stress, DNA Damage, and repair in four invertebrates by using lymphocytes from Atlantic cod (*Gadus morhua*) as a reference. Results indicated that invertebrate’s cells were more susceptible to oxidative stress induced DNA damage as compared to lymphocytes of *Gadus morhua* as well as no recovery observed in any species of invertebrates^63^. Yun *et al*.^18^ studied the electron beam induced DNA damage and repair in *S. litura* and reported that low dose of electron beam radiation resulted repair in damaged DNA but high dose of electron beam irradiation induced non-repairable DNA damage. The reason behind this might be a DNA repair capacity and cell proliferation rate. In invertebrates repair system is slight less efficient than vertebrates and very less difference in repair capacity leads to 10 to 20 fold more susceptibility to genotoxicants. On the other hand if cell proliferation rate is slow then the damaged cells were less frequently eliminated which might be a reason behind more damage and less repair.

At the end MTT assay was carried out on CHO cell lines. The results showed negligible toxicity of fungal extract on CHO cell lines which indicated its safety for mammals. Earlier cytotoxicity study by MTT assay was carried out on *Spodoptera litura* SL-1 cells to check the toxicity of destruxin A and it was observed that destruxin A was highly toxic for SL-1 cells^64^.

## Conclusion

In the light of above discussion it has been concluded that an endophytic fungus *Schizophylum commune* exhibited insecticidal activity due to its long term cytotoxic and genotoxic effects against *S. litura*. The study suggests the potential of *S. commune* as a biocontrol agent. Further there is need to conduct field studies to evaluates its efficacy under natural conditions.

## Materials and Methods

### Insect rearing

Larvae of *S. litura* were collected from the cauliflower fields around Amritsar (Punjab) India. The collected insect population was reared in laboratory under controlled temperature (25 ± 2 °C) and humidity (60–70%) conditions^11^.

### Isolation of fungi

Endophytic fungi were isolated from leaves and stems of healthy plants (Tulsi, Aloevera and Amaltas) collected from Amritsar (India). Collected samples were thoroughly washed with running tap water and sterilized sequentially with 70% ethanol for 2 min, 5% sodium hypochlorite solution for 5 min and then rinsed with sterile distilled water. Surface sterilization was confirmed by plating the water obtained after last wash. Each sterilized sample was cut into small pieces and placed on water agar plates supplemented with ampicillin (200 mg/ml) to suppress the bacterial growth. Plates were incubated at 30 °C till the emergence of hyphae. After emergence of hyphae, the hyphae tips were picked and cultured on Potato Dextrose Agar (PDA) plates. The culture was purified and maintained on PDA and stored at 4 °C^40^.

### Production of fungal extract

The production was carried out in 50 ml malt extract (malt extract = 20 g/l, dextrose = 20 g/l, peptone = 1 g/l, pH = 5.5) broth in 250 ml Erlenmeyer flask by inoculating one plug (1 cm square) taken from the periphery of an actively growing culture. The flasks were incubated at 30 °C and 250 rpm for 10 days. After 10 days extraction was carried out twice using ethyl acetate at 120 rpm and 40 °C. The extracts were concentrated by using rotavapor and dissolved in 1 ml Dimethyl sulfoxide (DMSO) and stored at 4 °C.

### Screening of fungi

Fifteen endophytic fungi were isolated and their insecticidal potential was screened. To screen the insecticidal potential of all isolated fungi, an artificial diet as recommended by Koul *et al*.^65^ with slight modifications was supplemented with ethyl acetate extract of all isolated fungal strains at a concentration of 500 µg/ml in 0.5% Dimethyl sulfoxide (DMSO). The second instar larvae (6 days old) were fed on control (0.5% DMSO) as well as on extract supplemented diet. The experiment was replicated six times with five larvae per replication. The larvae were observed daily. Among the all tested cultures of fungi, A4 induced maximum mortality. So A4 culture was selected for further studies.

### Identification of fungus

The active culture A4 exhibiting maximum insecticidal activity was identified on morphological and molecular basis. Slide culturing was done for morphological characterization using standard taxonomic keys^66^. For Molecular identification, DNA was isolated and amplification of the region covering ITS1-5.8S- rDNA- ITS2 was carried out by using forward ITS1 and reverse ITS4 primer. Purification and sequencing of DNA amplicons was performed at first base sequencing (Malaysia). The sequence similarity was matched with other available databases retrieved from NCBI using BLAST^67^.

### Phylogenetic Analysis

Alignment of retrieved sequences was done using ClustalW program. Phylogeny tree was constructed using Phylogeny in MEGA 7 program with 1000 bootstrap. Evolutionary history was inferred using neighbor-joining method. Evolutionary distance was computed using kimura-2-parameter model

### Bioassay studies

To evaluate the effect of A4 fungal extract on different biological parameters of *S. litura* different concentrations (125, 250, 500, 1000 and 2000 µg/ml) of fungal extract were made from stock solution having concentration 470 mg/ml in 0.5% DMSO and added in artificial diet. The Second instar larvae (6 days old) were reared on fungal extract amended diets as well as with control diet (0.5% DMSO) at controlled temperature 25 ± 2 °C and relative humidity 70 ± 5% conditions. The experiment was replicated six times with five larvae per replication. Each larva was put in separate container (4 × 6 cm) and the diet was changed daily till pupation. Different observations recorded were larval mortality, development period and percentage of adult emergence

### Oxidative stress, cytotoxicity and genotoxicity measurements

To evaluate the effect of A4 fungal extract on different parameters i.e oxidative stress, cytotoxicity and genotoxicity in *S. litura*, th*e* artificial diet having 276.542 µg/ml (LC50 value) concentration was made in 0.5% DMSO. The third instar larvae (12 days old) were reared on fungal extract amended diets as well as on control diet (0.5% DMSO) at controlled temperature 25 ± 2 °C and relative humidity 70 ± 5%. The experiment was replicated three times. For each treatment and control there are 10 larvae per replication. Each larva was put in separate container (4 × 6 cm) and the diet was changed daily. All these parameters were assessed using haemolymph of 12 days old larvae. For this haemolymph was collected from 10 larvae fed with same concentration and then it was pooled. The effect of fungal extract has been recorded on all above mentioned parameters after different time intervals (24 hr, 48 hr, 72 hr and 96 hr) using the following protocols: (a,b,c)

- Lipid peroxidation or malondialdehyde (MDA) content analysis:
  For lipid peroxidation estimation, collected pooled haemolymph was mixed with PBS (Potassium Phosphate buffer pH 7.0) containing 0.01% phenylthiourea. Haemocytes were removed from haemolymph by centrifuging it for 20 min at 10000 g at 4 °C and supernatant was used for MDA content determination. MDA content was assayed according to Jain and Levine^68^ with slight modifications that are incubation of supernatant at 95 °C with Thiobarbituric acid (TBA) and measurement of absorbance at 532 nm. MDA content was expressed as nanomole/ml by using 1.56 × 10^5^ M^−1^cm^−1^ extinction coefficient.
- Cell viability assay:
  For this acridine orange/ethidium bromide (AO/EB) double staining was used. Collected pooled haemolymph was stained with dye mixer (Acridine orange 100 µg/ml Sigma Chemical Co. and Ethidium bromide 100 µg/ml Sigma Chemical Co.). Finally stained cells were analyzed under fluorescent microscope at 400 × magnification. Viable cells emit green fluorescence and necrotic cells emit red fluorescence Er *et al*.^57^.
- Comet assay:

The DNA damage was quantified by alkaline single cell gel electrophoresis according to Yousef *et al*.^19^ and Yun *et al*.^18^ with slight modifications. For this collected pooled haemolymph (from 12 days old larvae) was mixed with phosphate buffered saline (PBS) [Sodium chloride (NaCl) 8 g/l, Potassium chloride (KCl) 0.20 g/l, sodium dihydrogen phosphate 11.50 g/l, potassium dihydrogen orthophosphate 0.20 g/l, double distilled water, pH 7.4] containing 0.01% Phenylthiourea (PTU) and layered on microscopic slides precoated with Normal Melting Point Agarose (NMPA 1% in PBS) after mixing with Low Melting Point Agarose (LMPA 0.75% in PBS), LMPA and haemolymph mixed slides were covered with cover slips and placed on ice. After solidification of agarose cover slips were removed and slides were placed in lysing buffer [2.5 M NaCl, 100 mM Ethylene diamine tetra acetic acid (EDTA), 0.25 M Tris aminomethane, 0.25 M Sodium hydroxide (NaOH), 1% triton X-100, 10% Dimethyl sulfoxide (DMSO), double distilled water, pH 10.0] for 2 hrs at 4 °C. After lysing slides were put in an electrophoresis buffer (1 mM EDTA, 300 mM NaOH, double distilled water, pH 13) for 20 mins to unwind DNA and then electrophoresis was carried out for 20 min at 25 V and 300 mA. At the end neutralization was done for 15 min by using neutralization buffer (0.4 M Tris amino methane, double distilled water pH 7.5). The slides were dried overnight and then stained with ethidium bromide (20 µg/ml) and analysed under a Nikon fluorescent microscope. DNA damage was assessed by using Casplab software.

### Recovery

After giving treatment of fungal extract at each time interval recovery of DNA damage was checked after 1 day and 2days by feeding treated larvae to control diet. Each exposure time (ET) has two recovery groups. i.e R1 = Recovery after 1 day, R2 = Recovery after 2days. The experiment was replicated three times. For each treatment there are 20 larvae per replication. After treatment at particular time duration these 20 larvae were fed with control diet, out of these 10 larvae were used to study recovery after 1 day and 10 were used to study recovery after 2days. Each larva was put in separate container (4 × 6 cm) and the diet was changed daily. The recovery of DNA damage was assessed by comet assay. Before checking recovery of DNA damage of each group, their lipid peroxidation profile and cell viability was also checked.

### Phytochemicals analysis

Detection of various phytochemicals viz phenolic, saponins, tannins, steroids, triterpenoids, glycosides, proteins was carried out by following the methodology of^69–72^. Total phenolic content (TPC) was determined by using Folin-Ciocalteu assay according to Hossain *et al*.^73^. For identification of phenolic compounds High performance liquid chromatography (HPLC) analyses were performed with Ultra-high performance liquid chromatography (UHPLC) (Shimadzu) system consisting of Nexera model. The mobile phase composed of 0.1 acetic acid and methanol. Flow rate was adjusted to 1 ml/min. The injection volume was 10 µl and the column was maintained at 40 °C and 2500 psi. The separation was achieved by a reversed-phase chromatographic using a C18 column (150 mm length and 4.6 mm diameter) having particle size of 5 µm. HPLC chromatograms were detected using a photo diode array detector (Dionex UVD 340U UV/VIS) at 280 nm wavelengths according to absorption maxima of analyzed compounds. Detection of phenolic compounds was carried out by comparison with standards. Each compound was identified by its retention time and by spiking with standards under the same conditions^12^.

### Safety evaluation of *S. commune* fungal extract by MTT assay on Chinese hamster ovary (CHO) cell lines

The *in vitro* cytotoxicity of *S. commune* fungal extract was assessed by using CHO cell lines by MTT (3-(4,5-dimethylthiazol-2-yl)-2,5-diphenyl-2*H*-tetrazolium bromide) assay. CHO cell line was procured from the National Centre for Cell Science, Pune, India. Cells were grown in Dulbecco’s modified Eagle’s medium supplemented with streptomycin (100 U ml^−1^), gentamycin (100 μgml^−1^), amphotericin B (0.25 μg ml^−1^) and 10% fetal bovine serum (Himedia) in a CO_2_ incubator (5% CO_2;_ 90% Relative Humidity) at 37 °C. 5 × 10^3^ cells were added in each well of 96 well plate and incubated at 37 °C, 5% CO_2_ for 24 h. The cells were treated with different concentration of *S. commune* fungal extract (1000, 500, 250, 125, 62.25 µg/ml) for 48 h, washed and 100 μl of fresh medium with 20 μl MTT solution (5 mg ml^−1^) was added in each well. The cells were incubated at 37 °C, 5% CO_2_ for 4 h. After incubation the medium was removed and formazan product was dissolved in 100 μl of DMSO (Dimethyl sulfoxide) and shaken for 10 min. The optical density was measured at 550 nm by microplate reader. Percentage of cell growth was calculated by using formula:

$${\rm{Percentage}}\,{\rm{cell}}\,{\rm{viability}}=({\rm{OD}}\,{\rm{of}}\,{\rm{treated}}\,\mathrm{cells}/\mathrm{OD}\,{\rm{of}}\,{\rm{control}})\times {\rm{100}}$$

### Statistical analysis

To study the effect of duration, recovery and to compare different concentrations while screening and bioassay studies one way analysis of variance (ANOVA) with Tukey’s test was performed and to study the effect of treatment student’s t-test was applied

### Availability of data and materials

All data generated or analyzed during this study are included in this published article and its additional files.

### Ethical approval and consent to participate

This article does not contain any studies involving human participants or animals performed by any of the authors.
